# The Innate Antiviral Factors APOBEC3G and APOBEC3H Interact with the Nucleocapsids of Human Coronaviruses in an RNA-Dependent Manner

**DOI:** 10.3390/v18080830

**Published:** 2026-07-28

**Authors:** Jordi Exposito Trivino, Alexandra Decloux, Margaux Renier, Théo Massart, Justine Petit, Maxence Collard, Kévin Willemart, Aurélien Sellier, Rodrigue Tesse, Samuel Kindylides, Jean-Claude Twizere, Charles Nicaise, Lionel Tafforeau, Nicolas A. Gillet

**Affiliations:** 1 Integrated Veterinary Research Unit (URVI), Namur Research Institute for Life Sciences (NARILIS), University of Namur, 5000 Namur, Belgium; jordi.exposito@unamur.be (J.E.T.); alexandra.decloux@unamur.be (A.D.); maxence.collard@unamur.be (M.C.); kevin.willemart@unamur.be (K.W.); aureliensellier118@gmail.com (A.S.); 2 Cell Biology Laboratory, Research Institute for Biosciences, Research Institute for Health Sciences and Technology, University of Mons, 7000 Mons, Belgium; margaux.renier@alumni.umons.ac.be (M.R.); theo.massart@umons.ac.be (T.M.); justine.petit@umons.ac.be (J.P.); rodrigue.tesse@alumni.umons.ac.be (R.T.); samuel.kindylides@sciensano.be (S.K.); lionel.tafforeau@umons.ac.be (L.T.); 3 Avian Virology and Immunology Unit (AVIVIR), Animal Infectious Diseases, Sciensano, 1050 Brussels, Belgium; 4 Viral Interactomes Laboratory, GIGA Institute, University of Liege, 4000 Liege, Belgium; jean-claude.twizere@uliege.be; 5 Molecular Physiology Research Unit (URPhyM), Namur Research Institute for Life Sciences (NARILIS), University of Namur, 5000 Namur, Belgium; charles.nicaise@unamur.be

**Keywords:** APOBEC3, APOBEC3G, APOBEC3H, SARS-CoV-2, coronavirus, nucleocapsid

## Abstract

Severe Acute Respiratory Syndrome Coronavirus 2 (SARS-CoV-2) evolution has been marked by the rapid accumulation of mutations, among which cytosine-to-uracil (C-to-U) transitions represent a major proportion of observed genomic changes. These mutations have been proposed to result from the activity of host APOBEC3 cytidine deaminases, innate immune enzymes capable of editing viral RNA. However, the molecular mechanisms underlying APOBEC3 involvement in SARS-CoV-2 biology remain poorly understood. Here, we systematically investigated physical interactions between APOBEC family members and the SARS-CoV-2 proteins using a Gaussia princeps protein complementation assay. Screening of APOBEC family proteins against the viral proteome identified specific interactions between APOBEC3G (A3G) and APOBEC3H (A3H) with the viral nucleocapsid (N) protein. These interactions were validated by co-immunoprecipitation and were found to be conserved across nucleocapsid proteins from all seven human coronaviruses, suggesting conserved structural determinants. Mechanistic analyses revealed that the RNA-binding and oligomerization capacities of A3G and A3H are key for their interaction with the SARS-CoV-2 nucleocapsid. Mapping experiments further showed that the C-terminal domain of N constitutes the minimal interacting region, with stronger binding observed in larger constructs encompassing adjacent regions, indicating cooperative stabilization. Further work will be needed to determine whether A3G and/or A3H can restrict viral replication and whether their interaction with the nucleocapsid allows them to access and mutate the viral genome.

## 1. Introduction

Severe Acute Respiratory Syndrome Coronavirus 2 (SARS-CoV-2) is a recently emerged zoonotic betacoronavirus belonging to the *Coronaviridae* family, characterized by an enveloped, positive-sense single-stranded RNA genome [1,2]. Since its emergence in late 2019, SARS-CoV-2 has continued to evolve, giving rise to multiple variants of concern. Over the past six years, the virus has adapted to preferentially infect the upper respiratory tract rather than the lower airways [3]. Its antigenic properties have also evolved, posing ongoing challenges for vaccine adaptation [4,5]. Understanding this evolution requires identifying the molecular drivers of viral diversification. The SARS-CoV-2 genome is replicated in the cytoplasm of the host cell thanks to a viral RNA-dependent RNA polymerase (RdRp). Unique amongst RNA viruses, coronaviruses encode a 3′ to 5′ exoribonuclease (nsp14) that act as a proofreading mechanism to correct for RdRp mispairing. Replication fidelity is tightly controlled and deficiency in those mechanisms lead to severe viral attenuation [6,7]. The massive sequencing effort conducted during SARS-CoV-2 pandemic allowed real-time tracking of mutations and emergence of variants. Importantly, genomic analyses have revealed an unusually high frequency of cytosine-to-uracil (C-to-U) mutations, accounting for about 30% of all single nucleotide variations [8,9]. Several authors have suggested that these mutations could result from the enzymatic activity of the innate immune effectors APOBEC3 [8,10,11,12].

Apolipoprotein B mRNA-editing enzyme-catalytic polypeptide-like 3 (APOBEC3) family contains seven members in humans: A3A, A3B, A3C, A3D, A3G, A3F and A3H. The APOBEC3 proteins are cytidine deaminases that convert C-to-U mainly on single-stranded DNA intermediates. Thus, these proteins restrict various viruses by mutating in their genome [13]. Herpesviruses, papillomaviruses, polyomaviruses, adenoviruses, hepadnaviruses and retroviruses are susceptible to C-to-U deamination caused by APOBEC3 proteins [14,15,16,17,18,19,20]. For example, the retrovirus HIV-1 is restricted by A3G that introduces mutations in the viral genome during reverse transcription [14,15]. Importantly, APOBEC3 proteins can also restrict viral replication by deaminase-independent mechanisms. For instance, A3G interacts with HIV-1 reverse transcriptase and impairs its processivity [21]. Within the APOBEC family, only APOBEC1 (A1), A3A and A3G have been shown to possess RNA editing activities [22,23,24,25,26] and A3A and A3G activities are less potent on RNA than on DNA. Thus, antiviral properties of the APOBEC3 proteins on RNA viruses (without DNA intermediates) are not definitively demonstrated. Depending on the extent and context of APOBEC3-induced mutations, the APOBEC3 can either inhibit viral replication or promote viral diversification and the emergence of new variants [27].

In coronaviruses family, several studies have suggested a potential role for APOBEC3 proteins in modulating infection. Thus, A3C, A3F and A3H were shown to restrict the human alphacoronavirus HCoV-NL63 in vitro, although the underlying mechanism remains unclear [28]. In 2022, Kim et al. expressed APOBEC1 (A1), A3A, or A3G in 293T cells along with a reporter generating 200-nt long SARS-CoV-2 RNAs, and detected site-specific C-to-U mutations in the synthetic viral transcripts [29]. Upon infection of Caco-2 cells expressing exogenous A1, A3A, or A3G, they observed that A1 and A3G did not affect SARS-CoV-2 replication, whereas, unexpectedly, A3A enhanced viral replication [29]. Nakata et al. performed a similar experiment infecting 293T cells expressing the SARS-CoV-2 receptor ACE2 (Angiotensin Converting Enzyme 2) and membrane protease TMPRSS2 (Transmembrane Serine Protease 2). They reported that only A3A (and no other APOBEC protein) was able to introduce C-to-U mutations in the viral RNA. They did not observed any significant impact of A3A exogenous expression on the viral titer [30]. Begum and colleagues reported that SARS-CoV-2 cannot sustain replication in THP-1 cells deleted for the A3A-to-A3G locus, suggesting of proviral role of certain APOBEC3 proteins [31]. Lastly, Li and colleagues expressed A1, AID (activation-induced cytidine deaminase), A3D, A3F, A3G, or A3H in Caco-2 cells. Following SARS-CoV-2 infection, they reported that APOBEC expression reduced viral genome levels while increasing the mutational burden of viral RNAs [32]. Overall, the role of APOBEC3 proteins in SARS-CoV-2 replication and evolution remains debated and no scientific consensus has been reached.

We postulated that if the APOBEC3 proteins do play a role in SARS-CoV-2 replication, they should interact with some of the viral proteins. Their identification should enlighten us regarding the role of the APOBEC3 proteins in SARS-CoV-2 replication. High throughput interactomic experiments have already been conducted and identified numerous interactions between viral and cellular proteins [33,34,35,36]. However, such screenings were conducted in cellular models using highly permissive cells with generally low to no expression of restriction factors. Thus, we undertook to quantify the interactions between the seven APOBEC3 proteins and the different SARS-CoV-2 proteins using *Gaussia princeps* complementation assay (GPCA). We observed that A3F, A3G and A3H interact with the viral nucleocapsid N protein. By focusing on the interaction between A3G/A3H and N, we showed that this interaction is greatly favored by RNA and dependent on A3G/A3H RNA-binding motifs. We also observed that the A3G-N interaction is mainly mediated by the first deaminase domain of A3G. More globally, we observed that the interactions between A3G/A3H and N are conserved across all seven human coronaviruses.

## 2. Materials and Methods

**Cell line.** Human embryonic kidney (HEK) 293T cells were maintained in Dulbecco’s Modified Eagle’s Medium (DMEM) supplemented with 10% Fetal Calf Serum (FCS) and 1% penicillin/streptomycin (PS). Cells were cultured at 37 °C in a humidified incubator containing 5% CO_2_.

**Plasmid construction.** Coding sequences of APOBEC proteins were amplified by PCR using Q5 High-Fidelity DNA Polymerase (NEB M0491). PCR primers introduced attB1 and attB2 recombination sites to enable Gateway cloning (primers sequences listed in Table S1). PCR products were first cloned (Gateway BP clonase II, ThermoFisher Scientific^®^, Waltham, MA, USA) into the pDONR223 Gateway donor vector to generate Entry clones. All entry clones were sequenced to assess the proper cloning of each APOBEC. These Entry clones were then transferred into Gateway pDEST plasmids (Gateway LR clonase II, ThermoFisher Scientific^®^) encoding either the Gluc1 fragment of *Gaussia princeps* luciferase for GPCAs, or an N-terminal Myc tag for co-immunoprecipitation experiments [37,38].

Open reading frames (ORFs) encoding SARS-CoV-2 Wuhan strain proteins and nucleocapsid (N) proteins from seven human coronaviruses (SARS-CoV-1, MERS-CoV, SARS-CoV-2/Wuhan/2020, HCoV-HKU1, HCoV-NL63, HCoV-OC43 and HCoV-229E), cloned into pDONR223, were obtained from C. Demeret (Pasteur Institute, Paris, France), and J-C. Twizere, (Uliège, Liege, Belgium). ORFs were subsequently transferred by Gateway recombinational cloning into pDEST vectors to generate constructs expressing either N- or SARS-CoV-2 protein-Gluc2 fusions for GPCAs, or 3 × Flag-tagged proteins for co-immunoprecipitation.

Negative controls consisted of three unrelated human ORFs (DBH1, LRRC and PLEKHA9) cloned into Gluc1-expressing plasmids.

Construction of nucleocapsid and APOBEC3G fragments. Nucleocapsid (N) fragments corresponding to five regions (N-arm, NTD, LKR, CTD and C-tail) were amplified by PCR using region-specific primers (Table S2). Primer combinations allowed the generation of all fragments shown in Section 3.4 (Figure 4B). PCR products containing attB1 and attB2 sites were cloned into pDONR223, verified by sequencing and transferred into pDEST vectors to express N-fragment-Gluc2 fusion proteins [37,38].

APOBEC3G CD1 and CD2 domains were amplified using primers listed in Table S3. PCR products with blunt ends were ligated using the KLD (Kinase-Ligase-DpnI) reaction mix (NEB). APOBEC3G W94A/W127A (A3G-Mut) and APOBEC3H W82A/W115A (A3H-Mut) expression plasmids were constructed by site directed mutagenesis using primers listed in Table S3.

Constructions were verified by sequencing.

***Gaussia princeps* luciferase protein-fragment complementation assay (GPCA).** For each screen, HEK-293T cells were co-transfected with 100 ng of each plasmid DNA using PEIMax^®^ (polyethylenimine, Polysciences). At 24 h post-transfection, cells were lysed and luciferase activity was measured using Renilla luciferase assay kit (E280, Promega, Madison, WI, USA), as previously described in [37]. When indicated, RNase (100 µg/mL; #EN0531, ThermoFisher Scientific^®^) was added to the lysis buffer and incubated 30 min at 23 °C prior luciferase activity reading.

Normalized Luminescence Ratio (NLR) was calculated for each pair at least in triplicate as described in [38]:

$$NLR=\frac{Gluc1\text{\_}A+Gluc2\text{\_}B}{\left(Gluc1\text{\_}A+empty\text{\_}Gluc2\right)+(empty\text{\_}Gluc1+Gluc2\text{\_}B)}$$

For GPCAs aimed at mapping APOBEC vs. SARS-CoV-2 protein interaction, three negative controls (DBH1, LRRC and PLEKHA9) were also screened against SARS-CoV-2 proteins-Gluc2 constructs. The threshold for positive interactions was set as follows: mean NLR of Neg-CTLR + 3 × SD.

For GPCAs aimed at mapping A3G-N interaction and assessing its conservation across human coronaviruses, only NLR values were used for comparisons. APOBEC3A served as the negative control, and all statistical analyses were performed relative to A3A.

Cell transfection and protein extraction for GPCA. HEK 293T cells were seeded in T25 flasks and transfected with 4 µg of pGluc1 or pGluc2 plasmid diluted in 250 µL OPTI-MEM. A PEIMax^®^ solution (30 µL of 1 mg/mL PEI) was mixed with the DNA solution and incubated for 30 min. The 280 µL transfection mix was added to the cells and incubated for 24 h at 37 °C.

Cells were harvested, washed with PBS, and lysed in 100 µL RIPA buffer (50 mM Tris-HCl pH 8.0; 150 mM NaCl; 1% NP-40; 0.5% sodium deoxycholate; 0.1% SDS) supplemented with protease inhibitors (2% cOmplete (Roche, Indianapolis, IN, USA), 10 µM PMSF) and 1% Triton X-100. Lysates underwent one freeze/thaw cycle, followed by sonication (5 cycles, 30 s ON/30 s OFF, high frequency, 4 °C). Samples were centrifuged 15 min at 15,000× *g* at 4 °C, and supernatants were collected.

Protein concentrations were quantified using the Pierce BCA Protein assay kit (ThermoFisher Scientific^®^), measuring absorbance at 550 nm. Before SDS-PAGE, 25 µg of protein were mixed with loading blue (SDS loading Buffer 5×) and heated at 94 °C for 3 min.

**Cell transfection for co-immunoprecipitation.** HEK-293T cells were transfected with 2 µg of expressing plasmid (combination of Myc-APOBEC coding sequences (AID, A3C, A3F, A3G, A3H) with 3xFlag-N, or empty vectors) using JetPrime (Polysciences, Warrington, PA, USA). Cells were collected after 24 h, washed with PBS, and lysed in 200 µL Co-IP buffer (50 mM Tris pH 7.5; 150 mM NaCl; 2 mM EDTA, 1% Triton X-100) supplemented with protease inhibitors (cOmplete, Roche). Lysates were incubated on ice for 20 min and centrifuged 15 min at 15,000× *g* at 4 °C. Protein concentration was determined using the Bradford assay (RotiQuant, Carl Roth, Karlsruhe, Germany).

RNase A treatment. After sonication and centrifugation, half of each sample was incubated with RNase A (100 µg/mL; #EN0531, ThermoFisher Scientific^®^) for 30 min at 23 °C.

Co-immunoprecipitation. Anti-Flag M2 Magnetic Beads (M8823, Sigma-Aldrich^®^, Saint Louis, MO, USA) were prepared according to the manufacturer’s instructions. For each sample, 500 µg of protein lysate was incubated with the beads overnight at 4 °C. Beads were washed two times with TBS and eluted either using sample loading buffer (for myc-A3H, AID, A3C). To this end, 20 µL of 2× loading buffer (125 mM Tris pH 6.5; 20% glycerol; 4% SDS; bromophenol blue) were added to the beads and incubated at 95 °C for 3 min. For A3G and A3F, elution was performed using acidic elution. Beads were incubated in 50 µL of 0.1 M Glycine-HCl, pH 2.8 at room temp for 5 min on a rotator. Eluted fractions were neutralized with 8 µL Tris-HCl 1 M pH 7.4. Out of these, 21 µL were collected and 7 µL SDS loading buffer 4x was added.

**Western blot.** Samples (35 µg of total lysate, or IP elutions) and protein ladder (#26616, ThermoFisher Scientific^®^) were loaded onto either 10% or 12% SDS-PAGE gels and then migrated for 1 h 30 at 120 V. Proteins were transferred to methanol-activated PVDF membranes for 2 h at 100 V/200 mA/50 W at 4 °C.

Membranes were blocked in TBS-T (Tris Buffered Saline supplemented with 0.05% Tween 20) and 5% bovine serum albumin (BSA) for 1 h and incubated at 4 °C with the appropriate following primary antibodies: rabbit anti-Gaussia princeps luciferase polyclonal (cat#PA1-181, ThermoFisher^®^ diluted 1/1000 in TBS-T and 5% BSA), rabbit anti-A3G monoclonal antibody (5210-87-13, cat# 12397, from Prof. Reuben Harris and obtained through the NIH AIDS Reagent Program, Division of AIDS, NIAID, NIH, diluted 1/1000 in TBS-T and 5% BSA), rabbit anti-HSP90AB1 monoclonal antibody (SAB4300541, Sigma-Aldrich^®^ diluted 1/1000 in TBS-T and 5% BSA), rabbit anti-C-Myc-tag (16236-1-AP, proteintech (rabbit) diluted 1/4000 in TBS-T and 5% BSA or 13-2500, Thermofisher (mouse), diluted 1/1000 in TBS-T and 3% BSA) and mouse anti-Flag Tag (MA191878, ThermoFisher^®^, diluted 1/1000 in TBS-T and 5% milk, or 66008-4-Ig, Proteintech (Rosemont, IL, USA), diluted 1/10,000 in TBS-T and 3% BSA).

After three washes for 5 min in TBS-T, membranes were incubated with the appropriate secondary antibodies for 1 h as follows: Swine anti-rabbit, swine anti-mouse Ig/horseradish peroxidase (HRP) (Agilent Technologies, diluted 1/2000), goat anti-rabbit HRP (31460, Thermo, 1/50,000) or goat anti-mouse (G21040, Thermo, 1/100,000). After three washes for 5 min, detection was performed using SuperSignal West (Pierce or Femto) substrates and imaged with the ImageQuant LAS 4000 system or Fusion FX7 (Vilber). Densitometry analysis was done using ImageJ software v1.54 [39].

**Statistical analysis.** Student’s paired *t*-test were used to compare means. *p*-values were calculated using paired comparisons.

## 3. Results

### 3.1. A3G and A3H Interact with the SARS-CoV-2 Nucleocapsid

**Figure 1:**
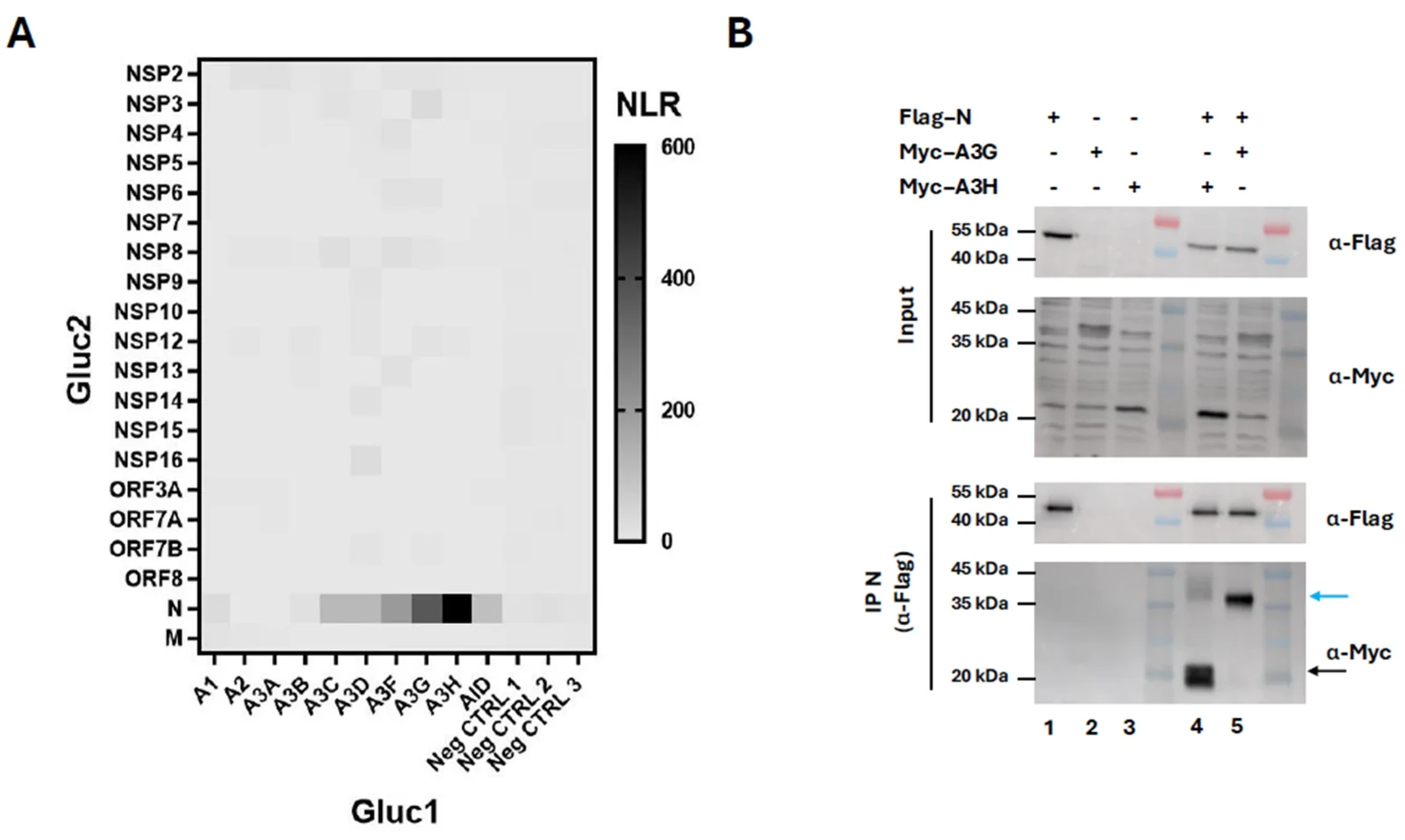
**A3G and A3H interact with the SARS-CoV-2 nucleocapsid protein.** (**A**) An interactome matrix between SARS-CoV-2 proteome and APOBECs using GPCA was conducted in HEK293T cells. Cells were co-transfected to express the human cytidine deaminases fused to Gluc1 moiety and the SARS-CoV-2 proteins fused to Gluc2. (**B**) Interactions between SARS-CoV-2 nucleocapsid and A3G and A3H were validated by Co-IP. HEK293T cells were transfected to express a Flag-tagged nucleocapsid protein together with Myc-tagged A3G or A3H. Nucleocapsid proteins were immunoprecipitated with beads flanked by anti-Flag antibody and the A3G or A3H were revealed by an anti-Myc antibody. A3G (blue arrow) and A3H (black arrow) co-precipitated with the SARS-CoV-2 nucleocapsid protein.

HEK293T cells were co-transfected with two plasmids: one encoding an APOBEC protein fused to the first half of luciferase (Gluc1) and the other encoding a SARS-CoV-2 protein fused to the second half of luciferase (Gluc2). If the proteins interact, a functional luciferase protein is reconstituted and light can be measured. APOBEC family contains 11 members, while the SARS-CoV-2 proteome comprises 28 proteins. To avoid false negative results, we only tested the fusion proteins that were validated by Western blot (Figures S1 and S2). The interaction map was generated using 10 APOBEC proteins and 20 SARS-CoV-2 proteins and the normalized luminescence ratios (NLR) were reported for each pair (Figure 1A). We observed that the NLR values for the nucleocapsid (N) protein and several APOBEC proteins (A3C, A3D, A3F, A3G, A3H and AID) are above the threshold for positive interactions (See material and methods) and the strongest signals were with A3G and A3H, with NLRs of 367 and 602 and a threshold value of 13.8.

To confirm the interaction between the SARS-CoV-2 N protein and A3G and A3H, we performed a co-immunoprecipitation assay. N was immunoprecipitated (Figure 1B, lanes 1, 4 and 5 in anti-Flag revelation), and A3G (blue arrow) and A3H (black arrow) were co-immunoprecipitated at a high level. Notably, the interactions between N and A3C, A3F or AID were also validated by co-IP (Figure S3).

### 3.2. RNA Favors the Interaction Between A3G and the SARS-CoV-2 Nucleoprotein

**Figure 2:**
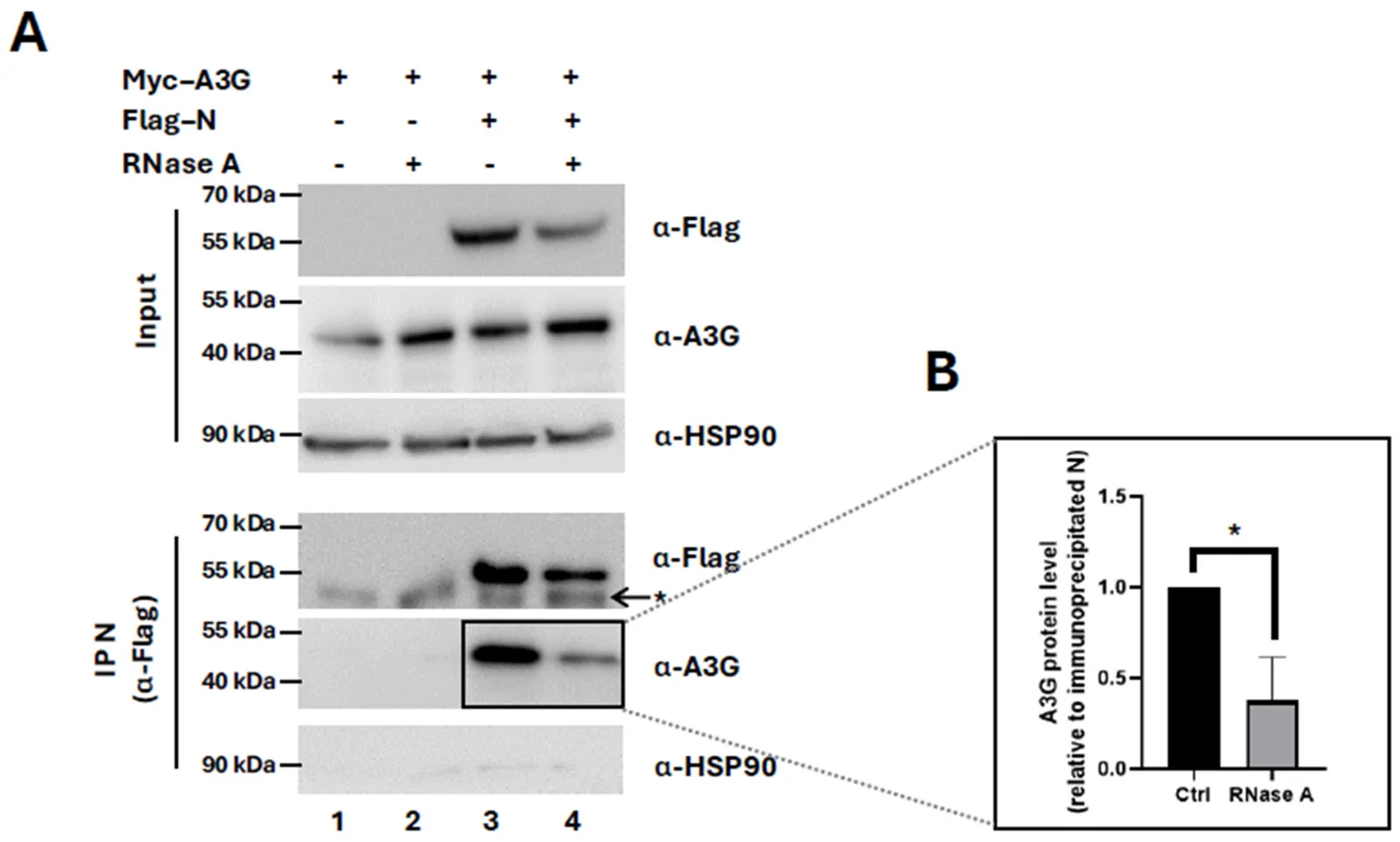
**RNA favors the interaction between A3G and SARS-CoV-2 nucleocapsid.** (**A**) HEK293T cells were transfected to express Myc-tagged A3G together with Flag-tagged nucleocapsid protein. Proteins were extracted and incubated with or without RNase A during 30 min at RT. Nucleocapsid proteins were immunoprecipitated with an anti-Flag antibody and A3G was revealed by an anti-A3 antibody. *: an unspecific band is present at 50 kDa, corresponding to the heavy chain of the antibody used for co-IP. (**B**) Relative A3G protein level immunoprecipitated with N in the presence of RNase A. Statistical analysis was performed with ratio *t*-test (* *p* < 0.05) on five independent experiments.

A3G and N both contain an RNA-binding domain. Since it was previously shown that RNA stabilizes the interaction between HIV-1 Vif protein and A3G [40] and also between A3G and SARS-CoV-1 N [41], we investigated whether it is also the case for SARS-CoV-2 N and A3G interaction. To investigate this, we performed a co-immunoprecipitation assay to examine the interaction between A3G and the N protein in the presence of RNase A. HEK293T cells were co-transfected as previously described and protein extracts were then incubated with or without RNase A prior to immunoprecipitation (Figure 2A). In the immunoprecipitated fraction, the presence of RNase A reduced the quantity of co-precipitated A3G (Figure 2B). These results suggest that the A3G-N interaction is at least partially RNA-dependent.

### 3.3. The RNA-Binding Capabilities of A3G and A3H Are Strongly Involved in the Interaction with N

We observed that RNA favors the A3G-N interaction. Because it is technically difficult to ensure that RNase treatment of the protein extracts achieves a complete degradation of the co-purifying RNAs, we cannot tell whether RNA is essential for the interaction or not. Interestingly, A3G is known to bind RNA via its N-terminal non-catalytic domain and substitutions of tryptophan 94 (W94A) and 127 (W127A) by alanines results in a complete loss of RNA-binding and oligomerization capacities [42,43,44,45,46]. Like A3G, A3H forms multimeric complexes with bound RNAs [47]. Similarly, tryptophan 82 and 115 are key for A3H binding to RNA [48]. Substitution of tryptophan 115 by alanine disrupts RNA-mediated dimerization of A3H yielding an RNA-free monomeric form [47].

**Figure 3:**
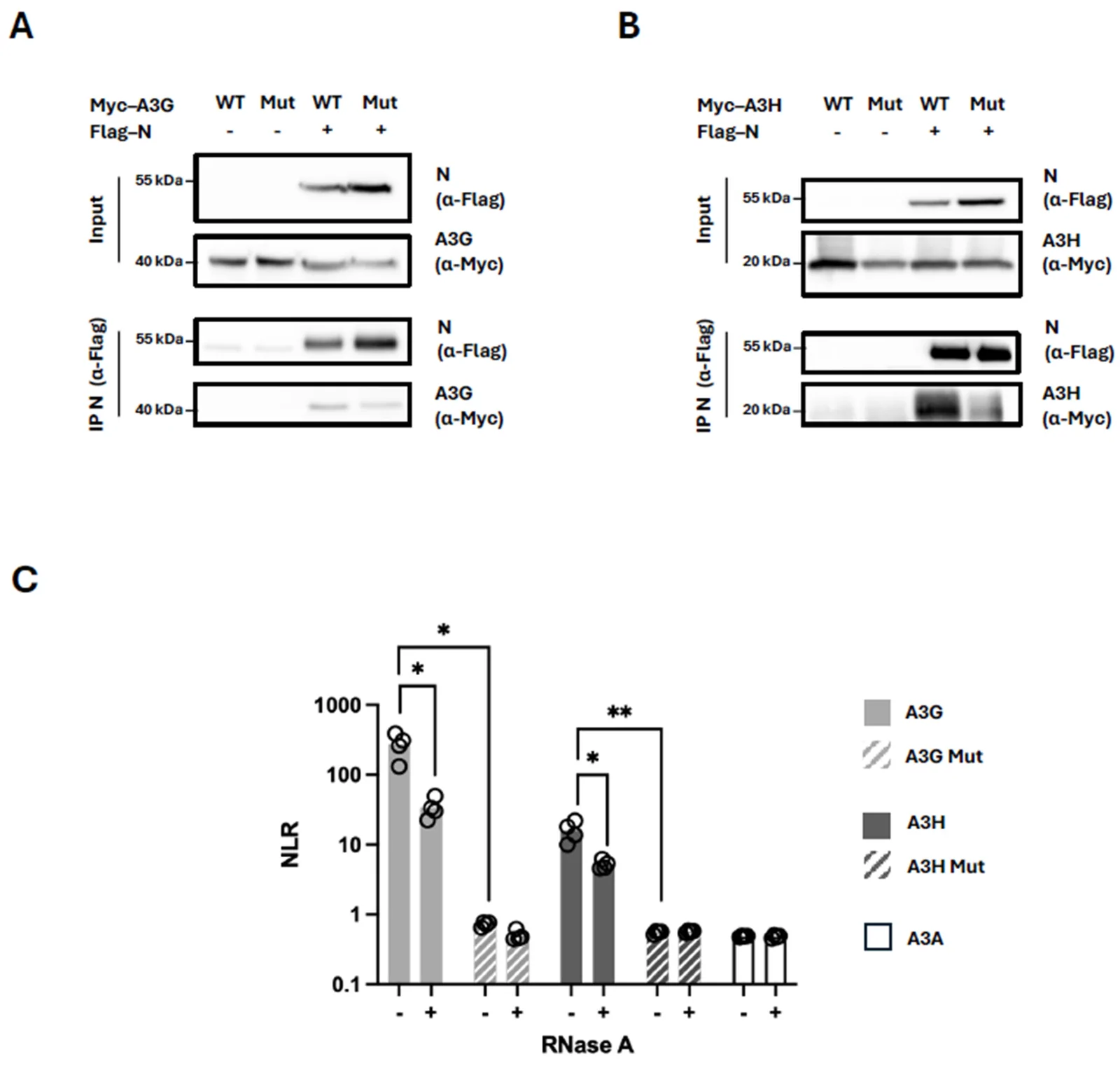
**The RNA-binding capabilities of A3G and A3H play a key role in the interaction with N.** (**A**,**B**) HEK293T cells were transfected to express Myc-tagged A3G (panel (**A**)) or A3H (panel (**B**)) together with Flag-tagged nucleocapsid protein. A3G Mut (W94A/W127A) and A3H Mut (W82A/W115A) lack RNA-binding and oligomerization capacities. Nucleocapsid proteins were immunoprecipitated with an anti-Flag antibody and A3G/A3H were revealed by an anti-Myc antibody. (**C**) Cells were co-transfected to express the SARS-CoV-2 N protein fused to Gluc2 moiety together with A3G WT, A3G-Mut, A3H WT, A3H-Mut or A3A (as negative control) fused to Gluc1 part. The cell lysates were incubated with or without RNase A during 30 min at room temperature prior luciferase activity reading. Statistical analysis was performed with *t*-test (* *p* < 0.05; ** *p* < 0.01). Number of independent experiments *n* = 4.

We performed a co-immunoprecipitation assay to test whether A3G or A3H mutants, unable to bind RNA and to oligomerize (A3G-W94A/W127A and A3H-W82A/W115A called respectively A3G-Mut and A3H-Mut), can still interact with SARS-CoV-2 N. HEK293T cells were co-transfected as previously described and the nucleocapsid protein was immunoprecipitated. We observed that the amounts of mutated A3G and A3H immunoprecipitated are lower compared to the WT version of the protein implying that RNA plays a key role for the A3G/A3H-N interaction (Figure 3A,B).

To better quantify the importance of the RNA-binding ability of A3G and A3H in the interaction with N, we performed GPCA between A3G, A3G-Mut, A3H and A3H-Mut and N. A3A was selected as negative control as it did not demonstrate significant binding with the full-length N protein (Figure 1A). The correct expression of the fusion proteins was verified by Western blot (Figure S1). We observed that RNase treatment reduces the signal both in A3G and A3H conditions (Figure 3C). Importantly, we observed that N-A3G_Mut and N-A3H_Mut signals are greatly decreased compared to N-A3G/A3H WT signals and are close to N-A3A, considered as the background (Figure 3C).

These results demonstrate that RNA and the RNA-binding capabilities of A3G and A3H play a major role in the interaction with the SARS-CoV-2 nucleocapsid protein.

### 3.4. A3G Interacts with the SARS-CoV-2 Nucleocapsid Protein Primarily via Its First Deaminase Domain

**Figure 4:**
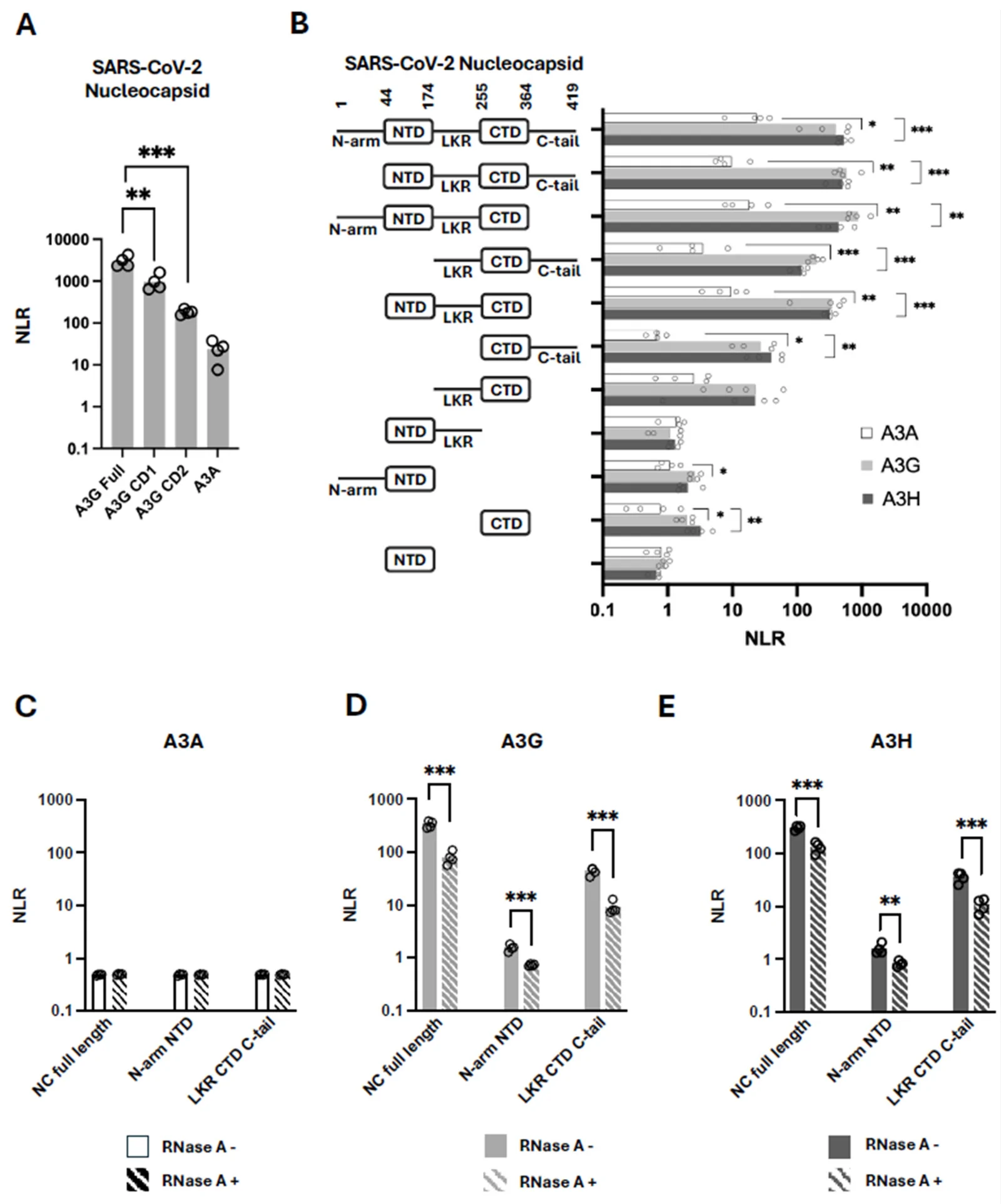
**Mapping interaction domain between N protein and A3G or A3H.** (**A**) A3G domains were assayed to map the interaction with N by GPCA in HEK293T cells. Cells were co-transfected to express the SARS-CoV-2 N protein fused to Gluc2 moiety together with A3A (used as negative control), A3G full-length, A3G CD1 (AA 1->196) or A3G CD2 (AA 197->384) fused to Gluc1 part. (**B**) Nucleocapsid domains (schematized on the left) were assayed to map the interaction with A3G and A3H by GPCA in HEK293T cells. Cells were co-transfected to express the different parts of SARS-CoV-2 nucleocapsid fused to Gluc2 and either A3A (used as negative control), A3G and A3H fused to Gluc1. (**C**–**E**) Impact of RNase treatment on the interaction between A3A, A3G and A3H with full or truncated versions of the SARS-CoV-2 nucleocapsid. Cells were co-transfected to express the different part of SARS-CoV-2 nucleocapsid fused to Gluc2 and either A3A, A3G and A3H fused to Gluc1. The cell lysates were incubated with or without RNase A during 30 min prior luciferase reading. Number of independent experiments *n* = 4. Statistical analysis was performed with *t*-test (* *p* < 0.05; ** *p* < 0.01; *** *p* < 0.001).

To determine which domains of A3G and N mediate this interaction, we performed a GPCA using different parts of the proteins. A3G has two structured domains: CD1 (residues 1–196) and CD2 (residues 197–384). CD1 domain is involved in the HIV-1 Vif-A3G interaction [49]. Furthermore, this domain is able to bind RNA [50]. HEK293T cells were co-transfected with Gluc2-full-length N and either with Gluc1-A3G CD1 or Gluc1-A3G CD2. The expression of each construct was verified by Western blot (Figure S4). The signal for the A3G-N interaction decreased significantly with A3G CD1 and CD2 domains compared to the full protein (Figure 4A). The decrease was greater with CD2 domain. This result suggests that the CD1 domain plays a more important role in the A3G-N interaction. Furthermore, the residues 104–156 have been identified as crucial for the interaction between SARS-CoV-1 N and A3G [41]. Given the similarity between these two viruses, particularly in terms of the N protein, it is not surprising that the same residues may be involved in binding with SARS-CoV-2 N. Of note, because A3H has only one deaminase domain, no truncation could be made.

Additionally, we wanted to determine which domain of N is involved in the interaction with A3G. N comprises two structured domains: NTD (residues 44–174) and CTD (residues 255–364) and three intrinsically disordered regions: N-arm (residues 1–43), LKR (residues 175–254), and C-tail (residues 365–419). The NTD domain binds to vRNA while the CTD domain is involved in homo-dimerization [51]. We thus tested which part of N interacts with A3G by GPCA. Thirteen N protein truncation mutants were generated and fused downstream of Gluc2 (Figure 4B). The expression of all constructs was verified by Western blotting (Figure S4). Constructs corresponding to the unstructured regions (N-arm, LKR and C-tail) were not detected and were therefore excluded from the analysis. Each N fragment was tested alongside A3A, A3G and A3H, fused to Gluc1. We observed that the smallest N protein fragment that interacted with A3G and A3H was the CTD domain (Figure 4B). A3G and A3H showed significant differences compared to A3A in the CTD-C-tail domain. The signal and difference were more pronounced with the LKR-CTD-C-tail. Overall, we found that larger and more complete N constructs exhibited stronger interactions with A3G and A3H. These observations suggest that the interaction is stabilized by cooperative contributions from multiple domains, particularly the LKR-CTD-C-tail region.

Finally, we examined the impact of RNase treatment on the interaction of A3G and A3H with different domains of N. RNase treatment reduced the interaction signal with full-length N but also with the N-arm–NTD and LKR–CTD–C-tail nucleocapsid fragments (Figure 4D,E). Although the CTD of N is not known to bind RNA directly, RNase treatment also weakened its interaction with A3G/A3H. These observations strengthen the idea that RNA-mediated bridging of the APOBEC3 proteins plays a major role in promoting their interaction with N.

### 3.5. A3G/A3H-N Interaction Is a Conserved Feature Among All Human Coronaviruses

**Figure 5:**
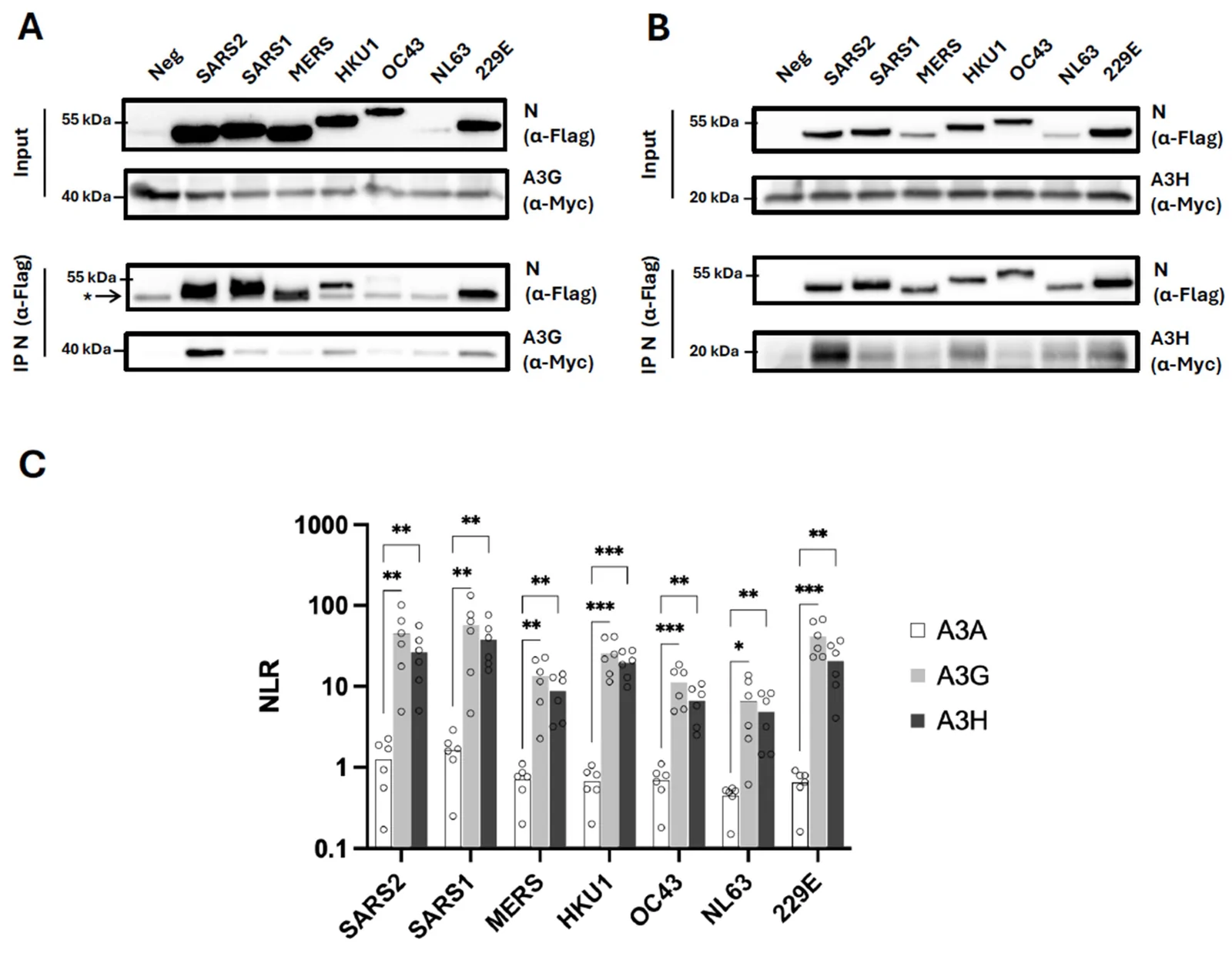
**A3G and A3H interact with the nucleocapsid proteins of all human coronaviruses.** (**A**,**B**) HEK293T cells were transfected to express a Flag-tagged nucleocapsid protein together with Myc-tagged A3G (panel (**A**)) or A3H (panel (**B**)). Nucleocapsid proteins were immunoprecipitated with beads flanked by anti-Flag antibody and the A3G or A3H were revealed by an anti-Myc antibody. *: an unspecific band is present at 50 kDa, corresponding to the heavy chain of the antibody used for co-IP. (**C**) HEK293T cells were co-transfected to express the seven human coronavirus nucleocapsid proteins fused to Gluc2 and either A3A (used as negative control), A3G and A3H fused to Gluc1. Statistical analysis was performed with *t*-test (* *p* < 0.05; ** *p* < 0.01; *** *p* < 0.001). Number of independent experiments *n* = 5.

Given the essential role of the N protein in binding and packaging the viral RNA genome, the question arises as to whether the interactions between A3G/A3H and N are conserved across all human coronaviruses. Although the amino acid sequence of the nucleocapsid is relatively conserved between the five betacoronaviruses (SARS-CoV-2, SARS-CoV-1, MERS-CoV, HCoV-OC43, HCoV-HKU1), this is less the case when comparing with the alphacoronaviruses’ nucleocapsid (HCoV-NL63, HCoV-229E) (Figure S5). These observations led us to hypothesize that the A3G/A3H-N interaction could be conserved in betacoronaviruses, but potentially absent in alphacoronaviruses. To investigate this, HEK293T cells were co-transfected with a plasmid encoding for the nucleocapsids of the different human coronaviruses together with a second plasmid encoding for A3G or A3H. We observed that A3G and A3H co-immunoprecipitate with the nucleocapsid of all human coronaviruses (Figure 5A,B). Similarly, we performed GPCA between A3G/A3H and N proteins from the different human coronaviruses. HEK293T cells were co-transfected with a plasmid encoding for Gluc2 fused to each N protein together with a second plasmid encoding for A3A, A3G or A3H fused to Gluc1. Expression of all N constructs was confirmed by Western blotting (Figure S6). A3G and A3H demonstrated interactions with all N proteins (Figure 5C). These results suggest that the A3G/A3H-N interaction is a conserved feature among all human coronaviruses, likely reflecting conserved structural determinants within the N protein.

## 4. Discussion

Since the beginning of the pandemic, the mutational profile of SARS-CoV-2 has been dominated by C-to-U substitutions, a trend that is still ongoing [8]. Although several in vitro studies point toward a role for APOBEC3 proteins [29,30,31,32], there is no consensus regarding their impact on viral replication (pro-versus antiviral), the specific APOBEC3 members involved, or the underlying mechanisms, including whether they target incoming genomic RNA and/or newly synthesized viral RNA.

In this paper, we tackle the question by testing whether APOBEC proteins can interact with SARS-CoV-2 viral proteins. Our interactome data are dominated by the interaction between N-A3G and N-A3H. Our findings, that A3G and A3H interact with N of SARS-2, are in line with several previous reports. Wang and colleagues reported that A3G co-immunoprecipitates with the SARS-CoV-1 nucleoprotein when expressed in 293T cells [41]. They reported that the region between residues 86 and 302 of the N protein (encompassing the NTD-LKR-CTD region) is key for its interaction with A3G [41]. Milewska and colleagues demonstrated co-immunoprecipitation of HCoV-NL63 nucleoprotein with A3C, A3F and A3H [28]. It is worth noting that the interaction between A3G and the nucleocapsid protein was not investigated by the authors, as A3G did not influence HCoV-NL63 replication in their in vitro assays. Using confocal microscopy, they reported some colocalization between A3C and HCoV-NL63 N [28]. Li and colleagues reported that SARS-CoV-2 N protein interacts with A1, AID, A3D, A3F, A3G, A3H, but not with A2, A3A, A3B, A3C and A4 when co-expressed in Hela cells [32]. Integrating our findings with previous studies, SARS-CoV-2 N consistently interacts with A3D, A3F, A3G, and A3H, but not with A2, A3A, or A3B. These observations may help pinpoint the residues involved in these interactions. The seven APOBEC3 genes originated from a common ancestral gene through duplication and rearrangement events [52]. APOBEC3 proteins contain one or two cytidine deaminase domains, also known as zinc-coordinating (Z) domains, which can be classified into three types (Z1, Z2, and Z3) based on characteristic amino acid motifs. Accordingly, A3A contains a single Z1 domain, A3C a single Z2 domain, and A3H a single Z3 domain, whereas A3B and A3G possess Z2–Z1 double domains and A3D and A3F contain Z2–Z2 double domains. The lack of interaction between A3B and SARS-CoV-2 N likely results from their different subcellular localizations; N being cytoplasmic whereas A3B is strictly nuclear [53]. The absence of interaction between A3A and N is more informative, because A3A is distributed cell-wide and could, in principle, bind N [53]. The comparison of the amino acid sequences of A3A (Z1 single domain) and A3H (Z3 single domain) and the construction of A3A-A3H chimeras could help to precisely identify the amino acids responsible for the interaction between A3H and N. Of note, in our work, we used the A3H hapII SV182 (most stable haplotype, splice variant counting 182 amino acids that localizes in the cytoplasm and in the nucleolus [53,54,55]).

In this work, we observed that interaction between A3G/A3H and N relies greatly on RNA and on A3G/A3H RNA-binding motifs. Of note, the nucleoprotein is known to promote liquid–liquid phase separation (LLPS) in the presence of the viral genomic RNA, forming biomolecular condensates to package the gRNA [56,57,58]. It would be interesting to test whether A3G/A3H can access those N-gRNA biocondensates thanks to their RNA-binding properties.

Even though it appears that the interaction of cytoplasmic APOBEC3 proteins with the nucleocapsid of coronaviruses is a conserved feature, the functional role (pro- and/or antiviral) of these interactions remains to be determined. Li and colleagues observed that, when SARS-CoV-2-infected cells were treated with sodium arsenite, A3G and N colocalize in stress granules and this relocation promotes vRNA mutagenesis [32]. They hypothesized that SARS-CoV-2 N protein acts as a bridging molecule, guiding host deaminases to target vRNA for editing, driving viral evolution [32]. However, it remains unclear whether viral RNAs (vRNAs) sequestered in stress granules can subsequently exit these structures and be packaged into viral particles. An alternative hypothesis is that N recruits A3G to stress granules to limit its editing activity on genomic RNAs destined for packaging. A similar mechanism has been described for HTLV-1 (Human T-lymphotropic virus type 1), in which the nucleocapsid protein binds A3G, sequestering it and preventing its incorporation into nascent virions [59].

The interaction between N and several cytoplasmic APOBEC3 proteins also raises question whether these proteins can be packaged in the virions. Wang and colleagues reported that A3G could be packaged in SARS-CoV-1 VLPs (through co-expression of A3G, N and M SARS-CoV-1 proteins in 293T cells) [41]. Incorporation of APOBEC3 proteins into virions is well established in HIV-1, where A3F and A3G are packaged in the absence of Vif [60,61]. However, the potential function of A3G within SARS-CoV-2 particles remains unclear, particularly whether it could influence translation or the initial round of replication of the incoming viral RNA. In this context, it would be informative to assess the impact of catalytically inactive A3G on SARS-CoV-2 replication. In that respect, Milewska et al. reported that enzymatically inactive A3C, A3F, and A3H retain partial antiviral activity against HCoV-NL63 [28].

Because A3G has been shown to interact with HIV-1 RT [62], we notably wonder whether APOBEC3 proteins could bind proteins of the SARS-CoV-2 replication transcription complex (RTC); namely nsp12 (RdRp), nsp13 (helicase), nsp14 (exonuclease), nsp10 and 16 (methyltransferases) and nsp7 and 8 (co-factors of the RTC). From our interactome data, no APOBEC protein directly interacts with RTC viral proteins although we cannot exclude undirect interactions, or interactions only when viral proteins are expressed in the context of an infected cell.

To date, in vitro studies investigating the role of APOBEC3 proteins in SARS-CoV-2 replication and evolution have primarily relied on cell lines overexpressing these enzymes. Establishing an in vitro system in which SARS-CoV-2 infection induces physiological levels of APOBEC3 proteins would provide more accurate insight into their true impact on viral biology.

## 5. Conclusions

SARS-CoV-2 evolution is marked by abundant C-to-U mutations potentially driven by APOBEC3 enzymes, but their role remains unclear. This study identifies conserved interactions between APOBEC3G/H and the human coronavirus nucleocapsid proteins, suggesting a possible mechanism by which these enzymes may access the viral genome.

## Data Availability

Data are contained within the article.

## Supplementary Materials

The following supporting information can be downloaded at: https://www.mdpi.com/article/10.3390/v18080830/s1. Figure S1: Detection of APOBEC- and negative control-Gluc1 fusion proteins. Figure S2: Detection of SARS-CoV-2 proteins fused with Gluc2 moiety. Figure S3: A3C, A3F and AID interact with the SARS-CoV-2 nucleocapsid protein. Figure S4: Detection of APOBEC3G domains-Gluc1 and SARS-CoV-2 nucleocapsid domains-Gluc2 fusion proteins. Figure S5: Homology between nucleocapsid proteins of all human coronaviruses. Figure S6: Detection of coronavirus nucleocapsid proteins fused with Gluc2 moiety. Table S1: List of primers used to construct APOBEC PCR fragment for Gateway cloning. Table S2: List of primers used to construct N truncated sequences PCR fragment for Gateway cloning. Table S3: List of primers used for A3G and A3H mutants.
