# Supplementary material for: The Innate Antiviral Factors APOBEC3G and APOBEC3H Interact with the Nucleocapsids of Human Coronaviruses in an RNA-Dependent Manner
Source: Viruses. 2026 Jul 28;18(8):830. doi: 10.3390/v18080830 (PMC13517127; doi:10.3390/v18080830)
Supplement: Supplementary file 1 [file viruses-18-00830-s001.zip › viruses-4254641-supplementary.pdf]

Supplementary Material

Figure S1

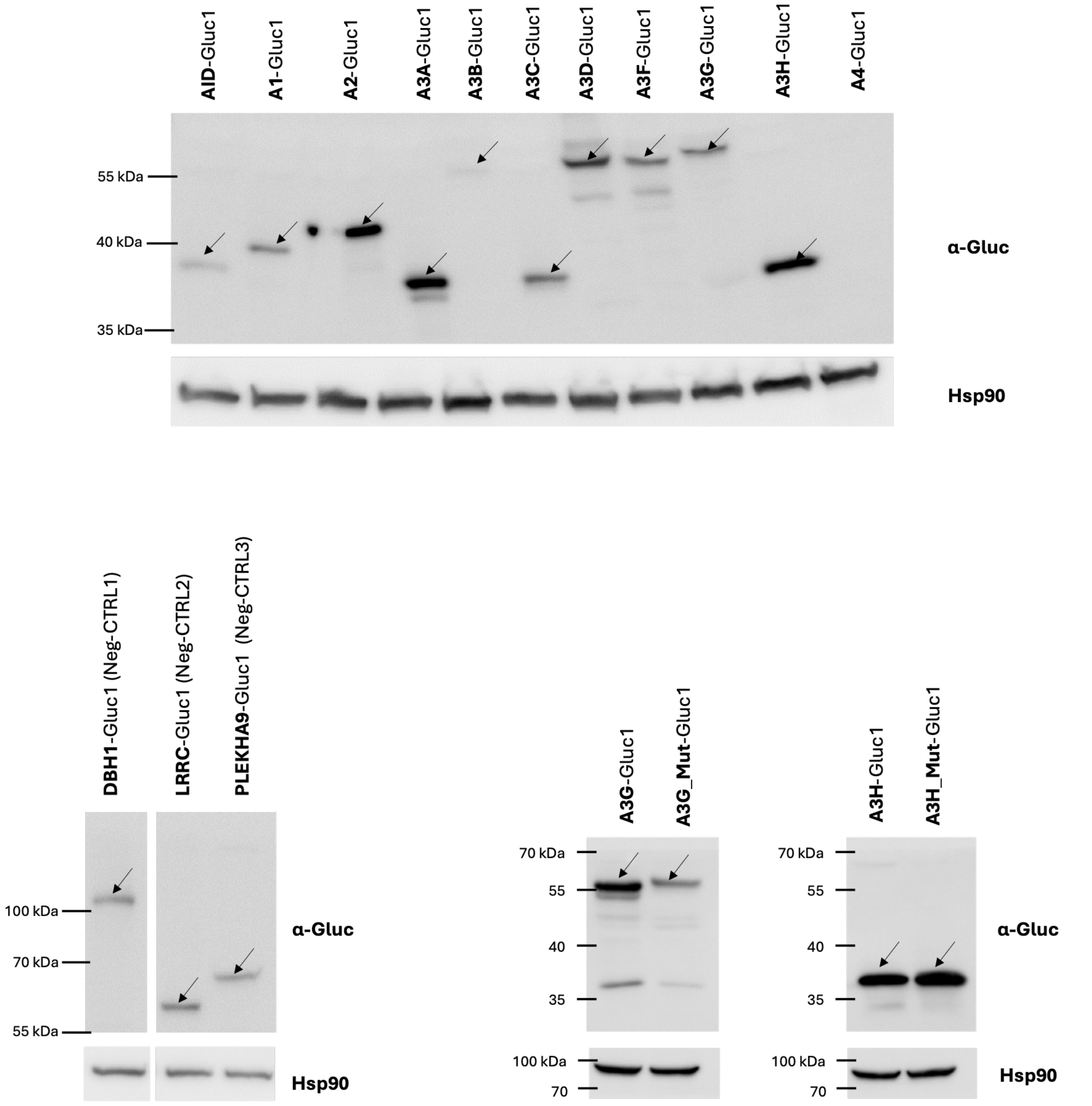

Figure S1: Detection of APOBEC- and negative control-Gluc1 fusion proteins.

HEK293T cells were transfected to express the various APOBEC proteins or negative control fused with the Gluc1 moiety at their C-terminal part and detected by Western blotting using an anti-Gaussia antibody.

Figure S2

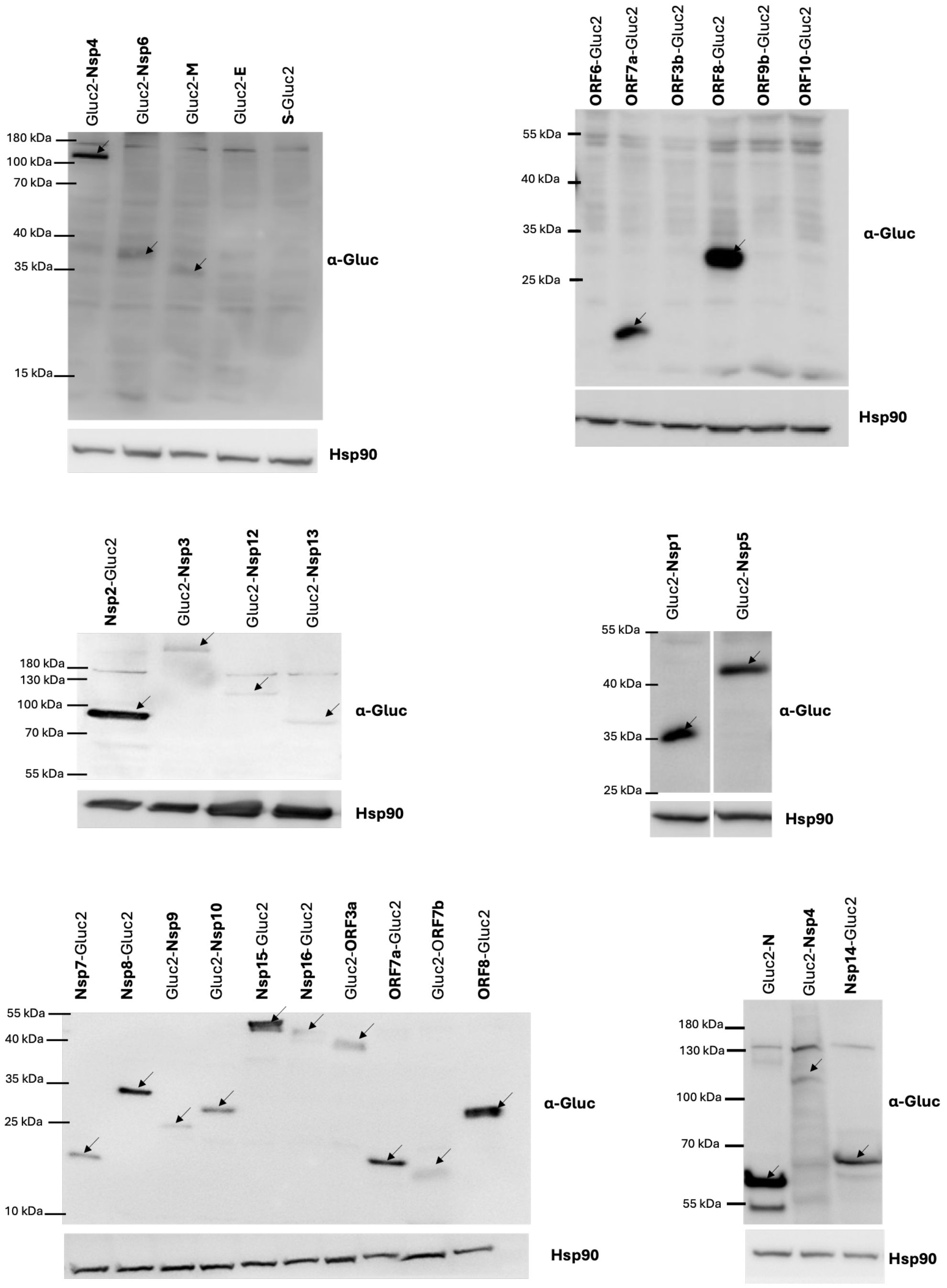

Figure S2: Detection of SARS-CoV-2 proteins fused with Gluc2 moiety.

HEK293T cells were transfected to express the various SARS-CoV-2 proteins fused with the Gluc2 moiety either in their N- or C-terminal part and detected by Western blotting using an anti-Gaussia antibody.

Figure S3

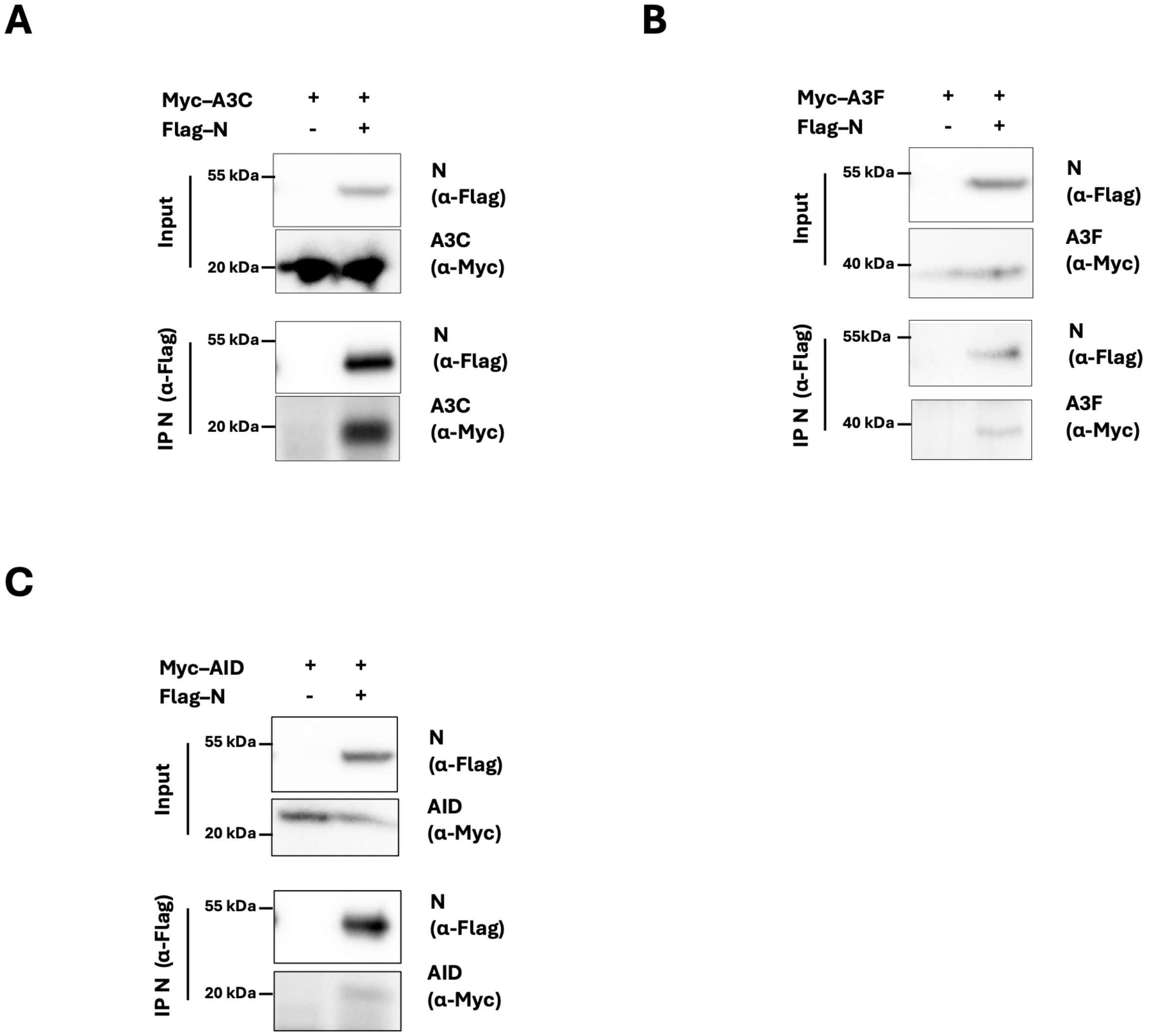

Figure S3: A3C, A3F and AID interact with the SARS-CoV-2 nucleocapsid protein.

HEK293T cells were transfected to express a Flag-tagged nucleocapsid protein together with Myc-tagged A3C, A3F or AID. Nucleocapsid proteins were immunoprecipitated with beads flanked by anti-Flag antibody and the A3C; A3F or AID were revealed by an anti-Myc antibody.

Figure S4

A

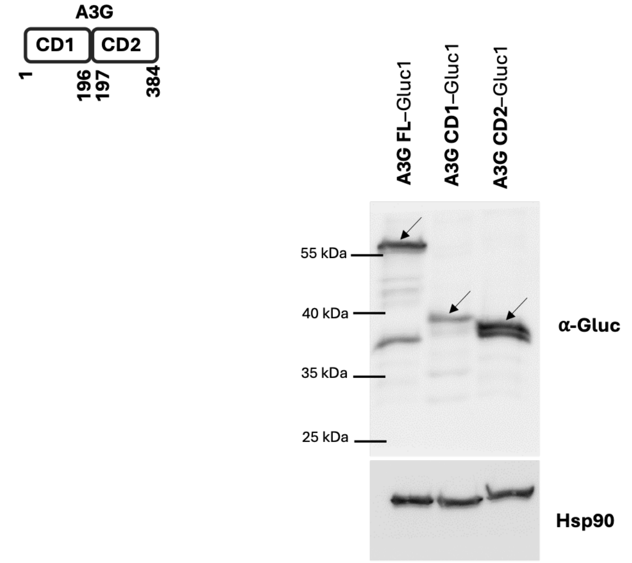

B

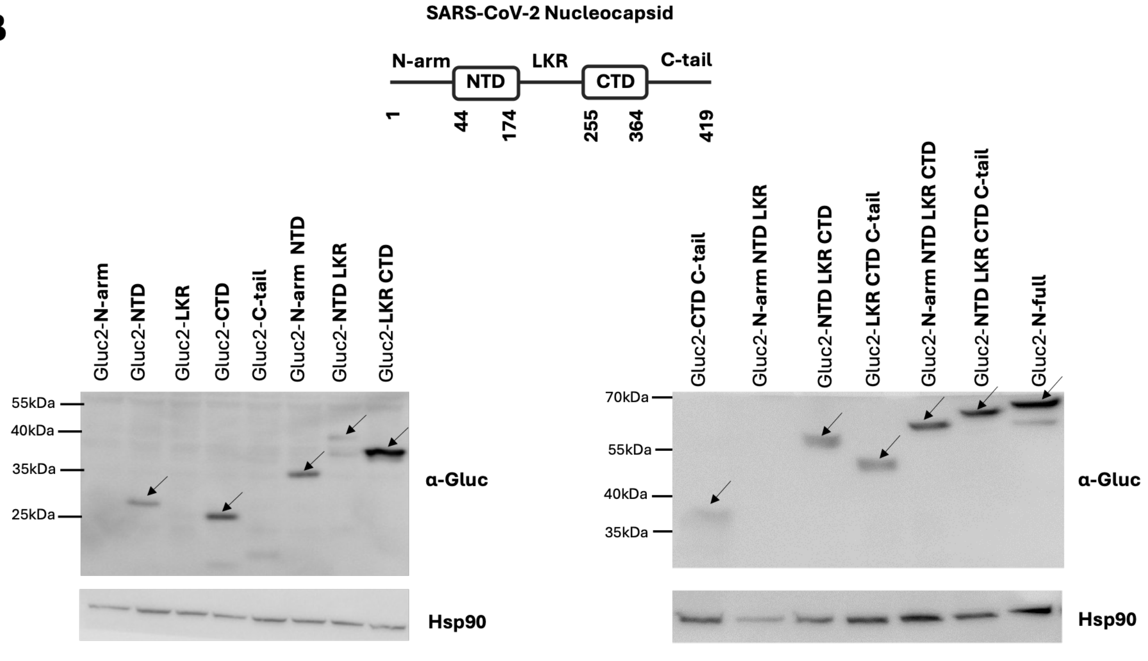

Figure S4: Detection of APOBEC3G domains-Gluc1 and SARS-CoV-2 nucleocapsid domains-Gluc2 fusion proteins. (A) HEK293T cells were transfected to express the different section of A3G fused with the Gluc1 moiety and detected by Western blotting using an anti-Gaussia antibody. **(B)** HEK293T cells were transfected to express the different section of SARS-CoV-2 N fused with the Gluc2 moiety and detected by Western blotting using an anti-Gaussia antibody.

Figure S5

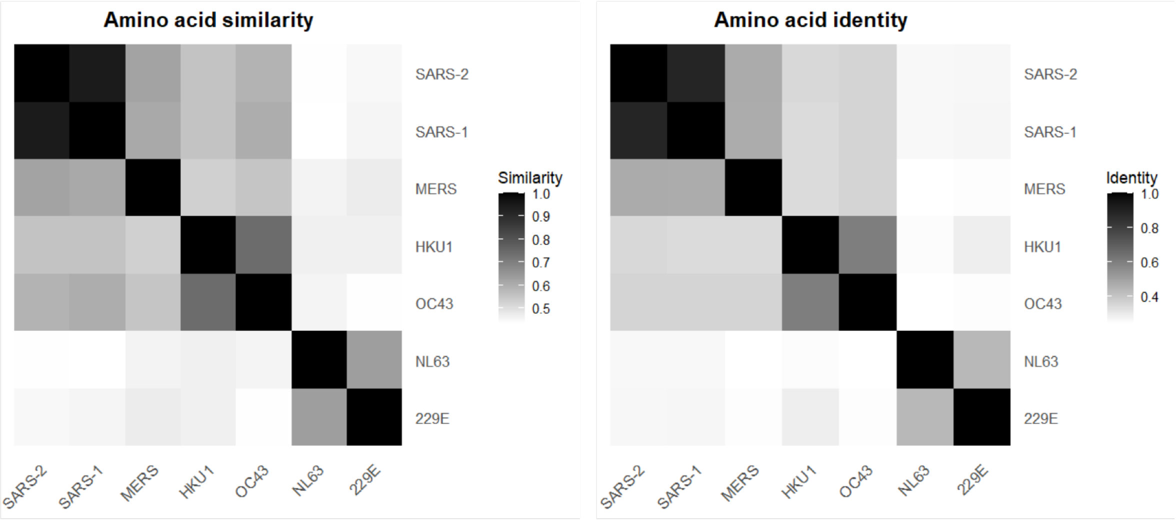

Figure S5: Homology between nucleocapsid proteins of all human coronaviruses.

Matrices show pairwise comparisons of amino acid sequences. The identity matrix represents the percentage of identical amino acids between two sequences, while the similarity matrix reflects the conservation of amino acid physico-chemical properties.

Figure S6

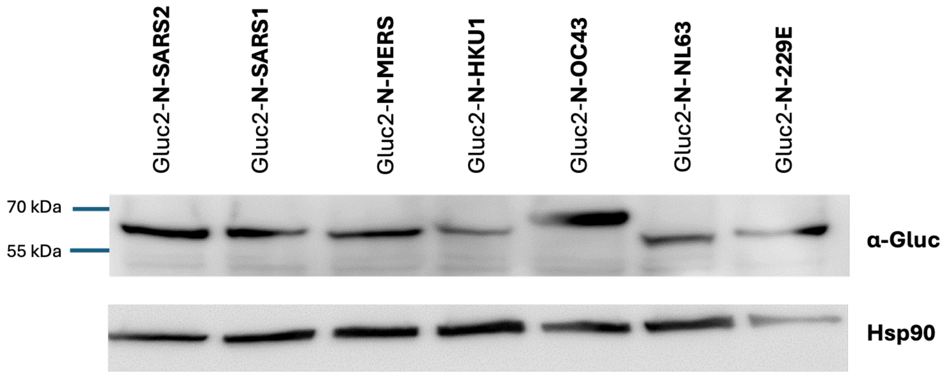

Figure S6: Detection of coronavirus nucleocapsid proteins fused with Gluc2 moiety.

HEK293T cells were transfected to express the various coronavirus nucleoproteins fused with the Gluc2 moiety and detected by Western blotting using an anti-Gaussia antibody.

Table S1: List of primers used to construct APOBEC PCR fragment for Gateway cloning.

Gene	Forward	Reverse	Annealing (°C)
APOBEC1	ggggacaactttgtacaaaaaagttggcATGAC TTCTGAGAAAGGTCC	ggggacaactttgtacaagaaagttggTCTCC AAGCCACAGAAGG	62
APOBEC2	ggggacaactttgtacaaaaaagttggcATGG CCCAGAAGGAAGAG	ggggacaactttgtacaagaaagttggCTTCA GGATGTCTGCCAAC	64
APOBEC3A	ggggacaactttgtacaaaaaagttggcatggaa gccagcccagcatc	ggggacaactttgtacaagaaagttgggtttccc tgattctggaagagc	67
APOBEC3B	ggggacaactttgtacaaaaaagttggcatgaat ccacagatcagaaatccg	ggggacaactttgtacaagaaagttgggtttccc tgattctggaagagc	65
APOBEC3C	ggggacaactttgtacaaaaaagttggcatgaa tccacagatcagaaacccg	ggggacaactttgtacaagaaagttggctggag actctcccgtagc	68
APOBEC3D	ggggacaactttgtacaaaaaagttggcatgaat ccacagatcagaaatccg	ggggacaactttgtacaagaaagttggctggag aatctcccgtagc	65
APOBEC3F	ggggacaactttgtacaaaaaagttggcatgaag cctcacttcagaaacac	ggggacaactttgtacaagaaagttggctcgag aatctcctgcagc	66
APOBEC3G	ggggacaactttgtacaaaaaagttggcatgaag cctcacttcagaaacac	ggggacaactttgtacaagaaagttgggttttcc tgattctggagaatgg	64

APOBEC3H	ggggacaactttgtacaaaaaagttggcatggctctgttaacagccg	ggggacaactttgtacaagaaagttggggactgctttatcctgtcaag	64
APOBEC4	ggggacaactttgtacaaaaaagttggcATGGAGCCCATATATGAGGAG	ggggacaactttgtacaagaaagttggTTTCTTCCCTTTCTTCTTCTTCTTTTC	64
AID	ggggacaactttgtacaaaaaagttggcATGGACAGCCTCTTGATGAAC	ggggacaactttgtacaagaaagttggAAGTCCCAAAGTACGAAATGCG	66

Table S2 : List of primers used to construct N truncated sequences PCR fragment for Gateway cloning.

Regions	Forward	Reverse	Annealing (°C)
N-arm	ggggacaactttgtacaaaaaagttggcATGTCCGACAACGGTCCCC	ggggacaactttgtacaagaaagttggCTGTGGCCTACGCTGCTTGC	54
NTD	ggggacaactttgtacaaaaaagttggcatgGGCCTGCCTAACAACACCG	ggggacaactttgtacaagaaagttggCTCAGCGTAGAATCCCTTGGGC	54
LKR	ggggacaactttgtacaaaaaagttggcatgGGCTCCAGGGGTGGAAGC	ggggacaactttgtacaagaaagttggGGCCTCAGCGGCAGACTTC	56
CTD	ggggacaactttgtacaaaaaagttggcatgTCTAAGAAGCCCAGGCAGAAGAG	ggggacaactttgtacaagaaagttggTGGGAAGGTCTTGTAGGCGTC	55
C-tail	ggggacaactttgtacaaaaaagttggcatgCCTACCGAGCCAAAGAAGGAC	ggggacaactttgtacaagaaagttggAGCCTGGGTGGAGTCAGCA	54

Table S3 : List of primers used for A3G and A3H mutants

Regions	Forward	Reverse	Annealing (°C)
CD1	CCAACTTTCTTGTACAAAGTGG	AATCTCCCCCAGCAAGATG	62
CD2	CTCAGACACTCGATGGATC	CATGCCAACTTTTTTGTAC	62
A3G_W94A	GTACATATCCgcgAGCCCCTGCAC	CAGGTGACCTCATACTC	60
A3G_W127A	CTACTACTTCgcgGACCCAGATTACC	AGGCGGGCAACAAAG	60
A3H_W82A	TTACCTCACGgcgAGCCCCTGCT	CAGGTGACTTGGTAGC	60
A3H_W115A	TACTACCACgcgTGCAAGCCCCAG	AGGCGGGAGGCGAAG	60
